# Improving Data Integrity in Samples Obtained From Web-Based Recruitment: Protocol for the Development of a Novel System for Assessing Participant Authenticity in a Remote Longitudinal Cohort Study of Polysubstance Use

**DOI:** 10.2196/69956

**Published:** 2025-08-14

**Authors:** Chavez R Rodriguez, Maya Campbell, Erin E Bonar, Jason E Goldstick, Maureen A Walton, Lewei A Lin, Lara N Coughlin

**Affiliations:** 1 Michigan Innovations in Addiction Care through Research & Education Program, Addiction Center Department of Psychiatry University of Michigan–Ann Arbor Ann Arbor, MI United States; 2 Injury Prevention Center University of Michigan Ann Arbor, MI United States; 3 Department of Emergency Medicine University of Michigan Ann Arbor, MI United States; 4 Center for Clinical Management Research VA Ann Arbor Healthcare System Ann Arbor, MI United States

**Keywords:** authentication, web-based recruitment, remote recruitment, research barriers

## Abstract

**Background:**

Remote recruitment for human participant research is increasingly popular due to its speed, cost-effectiveness, and accessibility for participants. However, in some cases, it can be particularly difficult to authenticate participants recruited remotely, which, unless adequately addressed, may pose a threat to data integrity and validity.

**Objective:**

This protocol aims to outline authenticity concerns encountered via remote recruitment for a longitudinal cohort study of adults reporting polysubstance use. Stemming from these concerns, we describe the development of a novel system of participant authenticity checks, designed with the goal of maximizing data integrity and minimizing the introduction of additional barriers to participating in the research. Finally, we examine rates of passing each active authenticity check among participants recruited via web-based advertisements.

**Methods:**

Participants were recruited through one of several modalities, including via electronic health records and a third-party company managing a web-based advertising campaign. All participants enrolled in the longitudinal study completed a screening survey, followed by a baseline assessment (involving a survey and an interview) before completing up to 4 weekly interviews and follow-up assessments at 4, 8, and 12 months after baseline. The authenticity check system described here was implemented for all participants recruited via web-based advertising. In addition to passive authenticity checks (ie, randomized online survey passwords), we describe a five-step active authentication protocol: (1) reviewing interest forms for duplication (interest form duplication review), (2) an attention check at screening (attention check), (3) reviewing personal information after completion of the screening survey for duplicates or inconsistencies (personal information verification), (4) a verbal identity confirmation at baseline (verbal identity confirmation), and (5) a review of participant responses for inconsistent reporting at baseline (consistent reporting review).

**Results:**

In total, 178 (6.85%) of the 2598 active authenticity checks administered were failed, leading to the exclusion of 119 unique potential participants due to fraudulent, inconsistent, or ineligible submissions. The 119 unique exclusions represented 11.13% (119/1069) of the potential participants identified via web-based advertising. Reviewing personal information provided at screening for inconsistencies (personal information verification) accounted for the largest number of failed checks (100/178, 56.2%), whereas reviewing interest form entries for duplicate personal information (interest form duplication review) yielded the fewest failures (7/178, 3.9%).

**Conclusions:**

The system presented provides an example of how researchers may increase confidence in the authenticity of participants recruited remotely, while avoiding the introduction of potential barriers to participating in research, such as requiring photo ID, online video call verification, or in-person verification. Such additional requirements for participants may systematically bias samples, especially when conducting research with populations that have been historically marginalized or those with stigmatized health conditions or behaviors.

**International Registered Report Identifier (IRRID):**

RR1-10.2196/69956

## Introduction

### Background

Use of fully remote or web-based methods for human participants research is becoming increasingly popular [1,2]. With a range of options, including phone- or SMS-based contacts, social media, or web-based advertising, either driven by the research team or through contracting recruitment companies that manage online advertisement campaigns [3], remote recruitment has been shown to be reliable and effective [4-7].

Remote recruitment provides study teams with several important benefits. First, these methods appear to be cost-effective, providing research teams with the ability to recruit relatively large samples without excessive expense [8-10]. Second, these large samples can be recruited quickly, such as by using mobile apps and websites [11] or via social media and web-based advertisements [8,12,13]. The ability to recruit geographically diverse samples that would otherwise not be feasible to recruit in person contributes to this speed and potential benefit [6]. Third, remote recruitment may simultaneously reduce the burden on the study team and reduce participant-level barriers for participating in the research [9,11,14]. For example, rather than requiring participants to travel to a physical laboratory space to determine their eligibility for a research study, research staff may instead administer screening assessments over the phone or via online platforms. Fourth, remote recruitment methods may be particularly effective tools for democratizing research participation among communities that have been historically excluded, including rural communities [15-17], racial or ethnic minority groups [18,19], sexual and gender minority groups [15,19,20], or individuals with stigmatized health conditions (eg, HIV and opioid use disorder) or behaviors (eg, substance use) [17-21]. Removing the requirement to participate in person removes a sizable barrier to participating, and the anonymity often afforded by remote participation can reduce biases based on social desirability [19].

Remote recruitment may be particularly effective for reaching groups that have been marginalized and stigmatized, such as participants who engage in substance use, including polysubstance use (PSU). Individuals with substance use disorders or who engage in PSU behaviors experience substantial stigma from health care providers [22,23] and the general public [24-26], which may reduce their trust in medical and research institutions. Remote recruitment methods may increase accessibility and help increase research representation among this population, as well as aid in recruiting members of rural or other underserved communities and individuals with stigmatized identities, who may face additional disparities in substance use disorder treatment. To effectively engage individuals reporting stigmatized PSU behaviors with remote recruitment, researchers must be able to quickly and accurately authenticate participants, without adding substantial barriers or burden to participation in the research that may systematically bias the sample.

Despite the potential benefits, remote or web-based recruitment may also pose several key limitations to study teams. First, remote recruitment can be inconsistent in terms of quality of responses, cost, and completion rates [14,27]. This variability may make it difficult for study teams to accurately estimate costs or require multiple remote recruitment avenues to ensure sampling goals are met. Second, it is considerably harder to verify a remote participant’s identity and authenticate that they are participating in good faith compared to traditional face-to-face recruitment [28-30]. Bots and fraudulent submissions via online platforms may be difficult to detect and require rigorous quality checks to preserve data integrity [28,31-35]. Across the literature, there are numerous examples of remote fraudulent submissions affecting data integrity, including among samples collected from crowdsourcing websites [36] and social media [37].

To combat these potential threats to validity, researchers have examined IP addresses [30,38-41]; compared names and email addresses to identify potential duplicate submission attempts [34,40]; required participants to enter study-designated passwords; updated web page identifiers to prevent repeated or automated responding [33,34,42]; or have implemented verification protocols for email addresses, phone numbers, and other forms of contact information to prevent multiple sign-ups with the same contact information [43,44]. In addition, studies sometimes capture similar information at multiple data points and then examine those responses for inconsistencies or unlikely responses [34,35,44,45] or use attention checks embedded within surveys to help ensure the validity of self-report data [46]. Although these strategies have been used across multiple study settings and populations, to the best of our knowledge, no studies have systematically implemented these checks as a means to enhance the rigor of remote recruitment methods when using third-party companies.

Remote recruitment companies have become increasingly popular and accessible to research teams, as evidenced by the multitude of options (eg, Prolific, Respondent, CloudResearch, User Interviews, Positly, and many others). Partnering with these companies yields many of the same advantages listed previously, along with the added benefit of outsourcing day-to-day management of the advertisement campaign to experts who may use data-driven tailoring to maximize visibility. However, little is known about the integrity of samples generated by these companies, especially considering the wide variety of options and approaches available. As evidenced by the increased popularity of new third-party companies, the remote recruitment space continues to rapidly change. Consideration of established remote authenticity strategies along with novel protocols may be necessary for research teams to adapt to the ever-changing authentication demands of remote research studies.

### Objectives

In this paper, we outline a novel system for authenticating participants recruited remotely via a third-party recruitment company for a longitudinal cohort study examining trajectories of opioid-involved PSU and other health outcomes. Given that individuals with PSU can experience marginalization from health care and research institutions, especially if they hold more than one stigmatized identities, this authentication process was also designed to avoid adding substantial barriers for potential research participants.

The aims of this paper are threefold: (1) to describe common participant authenticity concerns encountered by our research team during remote recruitment conducted using a third-party recruitment company; (2) to describe the gradual development and testing of a remote participant authentication check system put in place on an ongoing longitudinal cohort study of PSU trajectories, including 5 passive and 5 active steps; and (3) to examine rates of passing and failing each active check.

## Methods

### Study Methods

The ongoing longitudinal cohort study aims to better understand predictors and trajectories of opioid-involved PSU through 3 primary avenues. First, research suggests that there are individual-, social-, and community-level influences on substance use behaviors and overdose risk [47]. Synthesizing data at each of these levels may inform strategies for facilitating health care access and reducing overdose risk. Second, there are many motives for engaging in PSU [48], but research examining between- and within-person variation in these motives is currently lacking. Third, research suggests that alterations in reward and decision-making may be an important maintaining factor of substance use [49-51]. By examining individual decision-making and PSU choice preference through behavioral economics, the results of this study may inform the development and tailoring of interventions for PSU.

Variables of particular interest include quantity and frequency of substance use (eg, opioids, stimulants, cannabis, and alcohol), health care use, and experimental online tasks. Key covariates include demographic information, measures of mental and physical health, and barriers to accessing care. Participation lasts 12 months and consists of several surveys and interviews. Before continuing to the longitudinal portion of the study, participants complete a screening survey, followed by a baseline assessment (comprising a survey and an interview). During the longitudinal portion of the study, participants finish 4 intensive weekly interviews and then complete an assessment 4, 8, and 12 months after baseline. The selection of assessment time points served 2 key purposes aligned with balancing participant burden and staffing resources while being able to evaluate our research questions. First, the more intensive weekly interviews allow us to investigate episodic PSU in detail, given the recency of the assessments to the behaviors. Second, the 4-, 8-, and 12-month assessments were evenly spaced over 1 year to allow for consideration of longitudinal and longer-term trends in substance use patterns and health care use, as well as to maintain consistent contact with participants, facilitate retention, and minimize participant burden. Surveys may be completed online, over the phone, or in person (excluding the screening survey). Interviews include timeline follow-back (TLFB) assessments and must be completed over the phone or in person. Surveys and interviews both yield quantitative data. Interview-based TLFB assessments require participants to recount the events of a specific period and, using a calendar and these events to guide them, recall their alcohol and substance use during that period [52] to yield quantitative estimates of substance use. These interviews result in detailed data reflecting frequency and quantity of substance use consumption, categorically coded motives for use and routes of use, and information about the number of episodes of use a participant engaged in. Interview-based TLFB assessments have strong validity support for capturing detailed substance and alcohol use count data [52,53] and can be compared to survey-based measures of substance use for accuracy (details provided in Consistent Reporting Review section).

Out of 400 planned participants, as of October 16, 2024, the study team had enrolled 279 (69.8%) participants in the longitudinal portion of the study. Strategies to retain participants throughout the course of the study include providing monetary incentives for confirming follow-up assessment appointments, collecting multiple forms of contact information, and providing study activity reminders. We will impute data missing through skipped questions or attrition. We will investigate mechanisms underlying missingness to the greatest extent possible and evaluate the missing at random (MAR) assumption. If MAR is plausible, we will use multiple imputation by chained equations [54]. If the MAR assumption, which is not statistically checkable, is questionable, we will consider alternatives such as pattern-mixture models [55] or complete case analysis [56].

Eligibility criteria include past-month illicit opioid use or nonmedical use of a prescription opioid, use of another substance (ie, alcohol, cannabis, cocaine, crack, methamphetamine, ecstasy, lysergic acid diethylamide, mushrooms, or synthetic cannabis; nonmedical use of benzodiazepines or sedative anxiety and sleep medication; and nonmedical use of stimulant medication), and age between 18 and 75 years at screening. Eligible participants must also report having reliable access to a phone to complete study assessments and reside in the United States. Participants are excluded if they do not speak English or have medical conditions that preclude informed consent (eg, acute psychosis or severe cognitive deficits) as determined through a chart review (for participants recruited via electronic health records [EHRs]) or by research staff observation, including a brief cognitive impairment screening if required.

### Ethical Considerations

The study protocol was approved by the University of Michigan Medical Institutional Review Board (HUM00229563), including a University of Michigan participating site (HUM00234294). Waivers of Health Insurance Portability and Accountability Act authorization were requested and approved for recruitment and full study procedures. Waivers of documentation of informed consent were requested and approved for study consent to offer participants multiple options for providing consent (eg, self-administered online and verbally over the phone with a research staff member). All data presented in this report are deidentified, and this research is covered by a Certificate of Confidentiality from the National Institutes of Health.

Participants consent separately to participate in the screening survey and the longitudinal portion of the study. Study staff are available to answer questions about participation and ensure participants can describe the main components of the study, and participants are provided a copy of the longitudinal study consent form.

Participants are compensated US $15 for completing the screening survey, US $25 for each of the 4 weekly interviews, US $60 for the baseline assessment, US $60 for 4-month assessments, US $65 for 8-month assessments, and US $75 for 12-month assessments. To incentivize retention, participants can also earn up to an additional US $30 for confirming each study follow-up assessment (4, 8, and 12 months) appointment. Incentives are distributed as gift cards.

### Recruitment

Recruitment for this study has spanned several modalities. Methods have included using relevant diagnostic codes (eg, opioid use disorder) and other keywords to identify potentially eligible participants and then reviewing EHR, recontacting participants from other research studies focused on substance use, and in-person recruitment of inpatients at a large health care system. In addition, the research team has partnered with a third-party company to run a targeted, web-based advertising campaign. Advertisements appear on social media platforms, medical websites, and as part of Google Ads. Potential participants who click an online advertisement are directed to a landing page where they can briefly learn more about the study. The study team worded advertisements and the study landing page to mask specific eligibility criteria while still providing information about the purpose of the study. From the landing page, our team worked with our third-party partners to create an online interest form that allows interested individuals to answer several questions and provide contact information. Upon successful completion of the form, the individual’s contact information is provided to the study team on a private dashboard only accessible to the team, allowing research staff to initiate recruitment for eligibility screening.

Importantly, recruitment via the third-party company’s remote advertising campaign has presented challenges in participant authentication that were not commonly observed in other avenues of recruitment for this project (eg, EHR review). Duplicate contact information, the completion of several interest form entries from the same IP address within the span of 1 or 2 minutes, and inconsistent reporting from individuals recruited via this source necessitated additional protections for the integrity of data collected from remotely recruited participants. Consequently, the following passive and active authentication protocol was designed specifically for use on the remote advertising sample, and descriptive results reflect checks and findings only from this group (N=1069).

### Development of the Authentication Protocol

In the following authentication protocol, active checks are defined as those that involve direct effort and attention by a research staff member and result in a pass or a failure. Passive checks are those that are either automated or are study design decisions and do not directly result in a pass or a failure. Consequently, we present passing and failing rates only for active checks. The following protocol integrates strategies previously outlined in published work as well as novel authenticity checks.

In addition, as this protocol was developed during an ongoing study, some strategies were implemented earlier or later than others. Consequently, new checks were retroactively applied to participants who had already progressed past the intended checkpoint. This developmental process resulted in some instances wherein participants failed multiple active checks. Multimedia Appendix 1 includes the date each check was added to the study protocol.

### Passive Authentication Strategies by Third-Party Group

#### Overview

**Figure 1:**
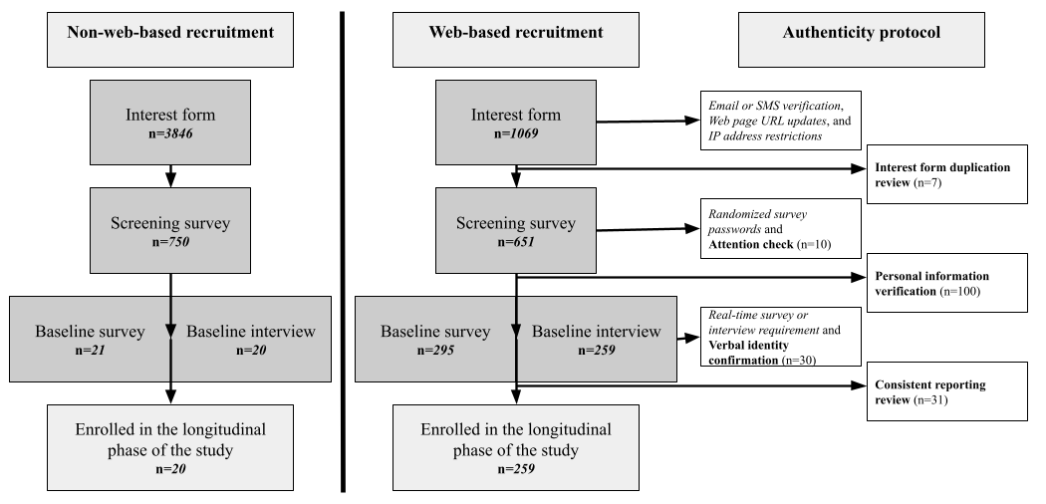
CONSORT (Consolidated Standards of Reporting Trials) diagram and authenticity checks administered as of October 2024. CONSORT diagrams for participants recruited via electronic health record or other non–web-based recruitment sources and for participants recruited via web-based advertisement are presented separately. The authenticity protocol is outlined for the web-based advertisement diagram. These diagrams do not list other reasons for ending participation not related to authenticity concerns, such as ineligibility or voluntary withdrawal. In the web-based recruitment diagram, arrows denote the timing of each authenticity check within the study flow. Italicized authenticity check names represent passive checks. Bolded authenticity check names represent active checks and include the number of failed checks resulting in withdrawal from the study.

Passive authentication strategies were implemented before individuals who completed the interest form were invited to complete screening for study eligibility. For each check, we present the primary authenticity concern the check was meant to address, as well as a brief procedure for implementation. Multimedia Appendix 1 provides a complete breakdown of this protocol. Figure 1 provides a diagram outlining when in the study flow these passive checks occur.

#### Email and SMS Verification

The purpose of the email and SMS check was to prevent bots from submitting interest forms, as well as to certify that an email address was valid and that the participant had access to the account. Our third-party partner is responsible for verification, which involves sending the individual an email with a unique link that they must click on within the next several minutes. Verification via the unique link constitutes a pass, whereas a lack of verification constitutes a failure.

Similar to email verification, participants must complete verification of their cell phone number via SMS text verification (given that the study inclusion criterion is access to any type of phone [eg, inclusive of landlines and other phones incapable of texting], this authenticity check adds an additional requirement to have a text-capable device. This decision was made for several reasons. First, participants recruited from nonremote sources can still participate without a text-capable phone. Second, the intention of providing a phone-based option to complete study assessments was to provide an avenue for participation for individuals without internet access. Given that participants recruited via web-based advertisements by definition have internet access, the study team considered the addition of this authenticity check acceptable to increase data integrity).

Our study involves several phone-based assessments, as well as texting and calling to confirm study sessions, making it essential for participants to have reliable phone access. Requiring SMS verification helps confirm that the phone number is real and connected to cellular service and that the participant has access to the phone.

After submitting their interest form, participants are texted a code by our third-party partners that they must enter into the study web page to demonstrate their access to that phone number. Without completing verification, participants’ interest forms are not completed and are not sent to the study team.

#### Web Page URL Updates

Working with our third-party partner, we changed the URL of our study landing page (from which potential participants could fill out an interest form) several times. Updating the landing page link reduces the likelihood that the interest form will be distributed in ways our study team cannot control (eg, via an online forum), which could reduce the quality of leads and increase study staff burden. Rather than being a pass or fail authenticity check, outdated versions of the study landing page are simply not accessible.

#### IP Address Restrictions

Early in recruitment, our team weighed the benefits of restricting interest form submissions by IP address. Although potential participants using the same internet network (eg, family members and roommates) would be unable to participate, we determined that the more pressing concern was that some individuals were completing multiple screening submissions under different names from the same IP address. Fraudulent submissions reduce the quality of interest forms, substantially increase study staff burden, and threaten data validity. Consequently, we opted to only allow a single interest form submission from each IP address. Attempts to submit additional interest forms are not processed. In addition to restricting interest form submissions by IP address, we also ensure that submission attempts from outside of the United States are blocked, given that US residence is an eligibility criterion.

Before automatic IP address restrictions were implemented, research staff completed a 1-time IP address review of all remotely recruited participants who had completed the interest form, including those who had already been screened. If multiple submissions were associated with a single IP address, study participants associated with all of those submissions were excluded. Individuals excluded for this reason are listed in Table 1 as part of the personal information verification active check. With the automation of these IP address restrictions, it was no longer necessary for staff to review this piece of information manually. Table 1 provides a breakdown of the total number of failed active authenticity checks. Table 2 provides a breakdown of the total number of unique participants withdrawn due to authenticity concerns.

**Table 1:** Number of active authenticity checks administered to and failed by potential participants recruited via web-based advertisements, as of October 2024^a^.

Check	Administered (N=2598), n (%)	Failed (n=178^b^), n (%)
Interest form duplication review	942 (36.3)	7 (3.9)
Attention check	517 (19.9)	10 (5.6)
Personal information verification^c^	424 (16.3)	100 (56.2)
Verbal identity confirmation	340 (13.1)	30 (16.9)
Consistent reporting review^d^	375 (14.4)	31 (17.4)

^a^Data are presented on the administration and rate of failure of active authenticity checks on the portion of our sample recruited via web-based advertisement. For the personal information verification and consistent reporting review, we present additional granularity on the reason for a failed check. This table provides the total number of checks administered and failed, inclusive of instances in which 1 individual failed multiple checks.

^b^Of the 2598 active authenticity checks administered, 178 (6.8%) failed.

^c^Reasons for failure of personal information verification include name consistency (33/100, 33%), duplicate contact information (14/100, 14%), address verification (43/100, 43%), and duplicate IP address (10/100, 10%; the study staff completed a 1-time review of duplicate IP addresses before this process was automated by our third-party partner; details in IP Address Restrictions section).

^d^Reasons for failure of consistent reporting review include date of birth (8/31, 26%) and data inconsistency (23/31, 74%).

**Table 2:** Number of unique participants recruited through web-based advertisements who were withdrawn due to authenticity concerns, as of October 2024 (N=119)^a^.

Number of failed checks	Participants, n (%)
5	1 (0.8)
4	2 (1.7)
3	6 (5)
2	30 (25.2)
1	87 (73.1)

^a^Data are presented on the number of unique participants who were withdrawn from the study due to failed active authenticity checks. These data represent only the portion of our sample recruited via web-based advertising.

### Passive Authentication Strategies Through Study Design

We included 2 additional passive authentication strategies through the design of the study that occur after individuals complete interest forms (Multimedia Appendix 1 provides details).

#### Randomized Survey Passwords

Because remote participants complete some study assessments online, each participant is assigned a unique identifier and password. Initially, passwords were the intended recipient’s unique identifier. However, early on in recruitment, our team noticed that some screening surveys were completed by individuals not assigned to a given identifier (eg, a 20-year-old man completing a screening survey using an identifier or password assigned to a 60-year-old woman). This was due to individuals guessing passwords to complete multiple screening surveys, which are remunerated US $15 for completion. To rectify the issue, our team began assigning each unique identifier a 10-digit password consisting of randomized strings, significantly reducing the likelihood that a password could be guessed and used by an unauthorized individual.

#### Real-Time Assessment Requirement

Built into our study design, all participants complete some real-time assessments (ie, over the phone or in person, with almost all participants thus far choosing the phone option). The baseline interview must be completed over the phone or in person, and participants can opt to complete the screening survey or baseline survey over the phone as well. Our team made this design choice to deter individuals from completing multiple submissions, as our team believed individuals would be less likely to submit fraudulent surveys if they knew the study would not be able to be completed entirely asynchronously.

### Active Authentication Checks

#### Overview

Once a potential participant completes the interest form, the study team receives their contact information and interest form results. Through the process of a 5-step active authentication protocol, study team members complete additional verification checks before a participant can be enrolled in the longitudinal portion of the study. We present each check, including the primary authenticity concern that the check addresses, the stage in the study flow when the check occurs, a brief protocol for implementing the check, and definitions of passed and failed checks. A failed check results in withdrawal from the study. Multimedia Appendix 1 provides a complete breakdown of the protocol. Figure 1 provides a diagram outlining when in the study flow these active checks occur.

#### Interest Form Duplication Review

Although passive checks prevent many duplicate or fraudulent interest form submissions, others are identified manually. Due to the multitude of recruitment streams on the project, it is possible that a participant recruited in person (eg, from the EHR) could also come across the online advertisement and mistakenly fill in the interest form. Furthermore, an individual could complete multiple interest forms using different names but with a duplicate phone number or address.

The interest form duplication review occurs after completion of the interest form and before participants are invited to complete the screening survey. For each new potential participant, research staff compare recruitment logs against the individual’s primary contact information and address. If the primary contact information or address is duplicate, the check fails and the individual is not invited to complete the screening survey.

#### Attention Check

If a submission passes interest form duplication review, the individual is invited to complete the screening eligibility survey. Several items on the screening survey require respondents to select yes or no regarding their substance use history. Through review of responses on the screening survey and baseline assessment, the study team noticed a pattern of affirmative answers to all substance use items on the screening survey, but at baseline, the same participant would report use of fewer substances during the same period. Consequently, the team added an attention check item to the screening survey in the section asking about substance use, which instructs the participant to select “no” on the item. Failing the attention check results in an end to participation, as this may indicate that a participant is inattentive or was selecting “yes” to all substance-related questions.

#### Personal Information Verification

Research staff then examine personal information reported by individuals who are eligible at screening, checking for inconsistencies before inviting them to complete the baseline assessment. Staff review the name, contact information, and address. Staff also reviewed the IP address before this process was automated. Comparing the name participants provide at the screening with the name associated with their interest form allows staff to identify surveys completed by someone other than the intended participant, as well as instances in which someone has guessed the password to a survey. Changes in contact information among individuals experiencing financial strain or housing instability may be common. However, duplicate contact information may indicate that an individual has submitted multiple interest forms, facilitating identification of potential fraudulent efforts to enroll in the study. Consequently, staff check contact information provided at screening against all recruitment records. If this newly provided contact information matches information entered in other study records, both records are withdrawn. For address review, we noticed that many individuals who completed the interest form provided incomplete or blank addresses. We believe that this tendency may be due to initial mistrust and that participants are more trusting of the study after they have interacted with a member of the study team (eg, received an email from a real person inviting them to complete the screening survey). Rather than comparing the address provided at screening to the one provided on the interest form, we chose to verify the existence of the address provided at screening using public online records. Determining that an address was nonresidential (eg, a bank) constituted a failed check unless a participant had previously indicated that they were experiencing housing instability.

#### Verbal Identity Confirmation

Once all postscreening survey authenticity checks are passed, participants are invited to complete the baseline assessment. Because the interview portion of the assessment must be completed over the phone or in person, participants must speak to a staff member before they are able to continue to the longitudinal portion of the study. The first time staff speak with a participant over the phone or in person, the participant must confirm their name, date of birth (DOB), and zip code before proceeding. This check identifies fraudulent submissions and inaccurate information and ensures that staff are speaking with the intended participant. If the respondent accurately provides the information when prompted, they pass the check. If the participant does not provide the correct information, refuses to answer, or does not provide the information when prompted (eg, hangs up and calls back later in the day to provide the information), they fail the check and are withdrawn from the study. For participants enrolled before this check was implemented, staff administered the identity confirmation check at their next phone-based or in-person assessment.

#### Consistent Reporting Review

After completion of the baseline assessment, at least 2 study team members (including at least 1 principal investigator [PI] or study coordinator) review a participant’s responses on all assessments for inconsistent reporting. First, DOB is reviewed for inconsistent reporting across study assessments. Research staff observed that submissions determined to be fraudulent often included discrepancies in reported DOB. Thus, DOB is assessed at screening and at baseline for comparison. Inconsistent reporting of DOB constitutes a failure and removal from the study.

In addition to DOB, at least 2 study team members (including at least 1 PI or study coordinator) also review responses on each assessment for indications of poor-quality responses. Poor-quality submissions may stem from fraudulent reporting or honest mistakes, but regardless, low-quality reporting is a threat to data validity. Research staff members may initiate this check if they observe highly unlikely or conflicting information reported by a participant. Examples of reasons to initiate this review include unusual routes of using substances (eg, snorting cannabis), significant and unexplained changes in substance use patterns over a very short time span (eg, reported using fentanyl every day in their screening survey but reported no lifetime opioid use in their baseline survey), or other cues from interactions with the participant (eg, participant A references an email sent to participant B as if the email had been sent to participant A).

Baseline inconsistency reviews begin with examination of the dates of the screening survey and baseline assessment to establish which items, based on response window (eg, past week, past year, or lifetime use), are expected to overlap. Next, research staff review each response to items with overlapping time frames from the screening and baseline surveys. For example, if a participant reported no lifetime use of cannabis in the baseline survey but then reported weekly cannabis use in the baseline interview completed 2 days later, that would be recorded as an inconsistency. Of course, it is reasonable that a participant could make errors in reading and answering survey questions; therefore, we set a threshold for the number of inconsistencies required to prompt the next stage of review. Specifically, if it is determined that there are ≥4 inconsistencies across the screening survey and baseline assessment, the research staff member prepares a formal report of all potential inaccuracies for the project PIs and coordinator to review. The PIs and coordinator examine all inconsistencies to determine if any can be explained more parsimoniously. If the PIs and coordinator deem at least 4 inconsistencies to be due to poor-quality reporting (ie, not otherwise reasonably explainable), the check is considered failed and the participant is withdrawn from the study. If ≤3 inconsistencies are deemed to be due to poor-quality responses, the check is passed and the participant may continue in the study.

The criterion of 4 inconsistent responses was based on a preliminary review of data from participants who had passed all other checks (ie, high likelihood that they were genuine and eligible participants). Among these authenticated participants, the maximum number of inconsistencies was 3.

### Data Analyses

We computed descriptive findings (eg, frequencies and proportions) regarding the active authentication system, including passing rates for each.

## Results

Non–web-based recruitment for this study began in fall 2023. Web-based recruitment, facilitated by a third-party recruitment company, began in January 2024. Results of the recruitment process are presented through October 2024 in Figure 1, divided by recruitment source (web-based recruitment vs non–web-based recruitment). A timeline of the date each strategy was implemented can be found in Multimedia Appendix 1. Of the 4915 interest forms completed, 1069 (21.75%) were completed by participants recruited remotely using web-based methods. Of the 1401 screening surveys completed, 651 (46.47%) were completed by participants recruited remotely using web-based methods. A total of 259 participants recruited using web-based methods were eligible and completed the baseline assessment, resulting in 39.8% (259/651) of the participants screened from this recruitment method completing the baseline assessment and continuing to the longitudinal portion of the study. A total of 20 participants recruited from non–web-based recruitment sources were eligible and completed the baseline assessment, resulting in 2.7% (20/750) of the participants screened from this recruitment method completing the baseline assessment and continuing to the longitudinal portion of the study. Of the 279 participants currently completing the longitudinal phase of the study, 259 (92.8%) were recruited using web-based methods.

The authentication protocol has been applied to the 1069 individuals completing interest forms who were recruited via web-based avenues managed by a third-party recruitment company. The total number of active authentication checks administered to this sample as of October 2024 is presented in Table 1, along with the number of failed checks. Altogether, 2598 active authenticity checks were administered to the 1069 individuals recruited remotely. In total, 6.85% (178/2598) of those checks failed. Because some checks were applied retroactively, individuals could fail multiple checks (Table 2 provides a complete breakdown). Consequently, 11.13% (119/1069) of unique individuals recruited by web-based methods were withdrawn from participation due to failed checks.

The largest source of failed active checks was personal information verification, which comprised 100 failures. Of those 100 failures, most were due to either providing a different name on the screening survey than on the interest form (n=33, 33%) or providing a nonresidential address in the absence of reporting housing instability (n=43, 43%). The consistent reporting review and verbal identity confirmation accounted for the second and third largest sources of failed active checks, comprising 31 and 30 failures, respectively. Alternatively, the interest form duplication review had the lowest rate of failure at 0.7% (7/942) and accounted for the fewest failures.

## Discussion

### Summary

This report describes the development and initial implementation of authenticity checks for remote recruitment procedures in a sample of individuals reporting opioid-involved PSU. We discuss findings and conclusions related to the success of web-based recruitment methods for this population, the utility of the authenticity protocol designed for this study, the use of third-party web-based recruitment companies for conducting human participant research, and avoiding the introduction of barriers to participation when choosing and implementing authentication systems.

### Web-Based Recruitment

Consistent with previous work examining the utility of web-based recruitment methods in substance use research, the preliminary results of our investigation suggest that these methods are effective [17,21]. In addition, these methods appear to be considerably faster than non–web-based recruitment avenues [8,12,14]. Despite far fewer completed interest forms compared to those recruited via non–web-based methods, the vast majority of our sample has been recruited from a web-based advertisement campaign managed by a third-party recruitment company. There are several possible reasons for this success. First, as has been observed in previous studies, web-based recruitment has the benefit of reaching a much larger pool of possible participants (eg, national recruitment) [6]. This breadth, combined with the tailoring of our advertising campaign, likely contributed to the high rate of eligible participants observed in the data. Second, remote research methods have been shown to be effective when recruiting participants based on stigmatized health behaviors, including substance use [17,21]. Third, participants responding to targeted advertisements are self-selecting into the study, whereas reaching participants through some non–web-based methods, such as EHR review, requires the study team to initiate contact with the participant.

### Takeaways From the Authenticity Protocol

The finding that study design and automated integrity protocols (eg, IP address verification) alone were not sufficient for deterring fake or fraudulent submissions is consistent with the recommendation to use both active and passive authentication strategies [30]. Considering the passive strategies, research suggests that email or SMS verification [43,44], IP address review [30,40], and requiring participants to input unique passwords each contribute to increasing data integrity [33,34]. It was highly feasible to implement these passive strategies, primarily due to the services provided by the web-based recruitment company.

Regarding the active authenticity checks, the interest form duplication review was considerably less efficient in this study compared to previous research [34,40]. As presented in Figure 1, this active check occurs after participants have already completed email and SMS verification and met IP address restrictions. The finding that very few potentially fraudulent submissions were detected with this check may be evidence that these passive strategies have been effectively dissuading or detecting these submissions, thus decreasing the necessity of checking interest forms for duplicate entries.

Although the attention check during screening identified very few instances of inattentive or automatic responding, the ease with which such a check can be added to a survey-based assessment adds to its utility as part of an authentication protocol. There are several possible explanations for the very low failure rate. First, it is possible that the attention check was too simple and did not sufficiently distinguish between attentive and inattentive or automatic responding. We used an instructed response item as an attention check [46], so it is possible that different attention check items could result in different failure rates. Second, it is possible that most inattentive participants or those using automatic response patterns were prevented from continuing in the study due to the previous passive strategies.

Similar to the findings of other studies [34,40], assessing similar constructs at different time points and examining responses for consistency (ie, personal information verification) appeared to be a particularly effective authentication strategy. Because it is possible that some participants, especially those who may have had negative previous interactions with research teams or who were concerned about the possibility of internet data breaches, did not provide entirely accurate information on the interest form due to a lack of trust, we recommend assessing these demographics once research teams have more personalized interaction (eg, email, phone, or in-person contact). In addition, in some instances, participants may make typographical errors or misremember personal information, so it is important to have staff carefully review and understand verification procedures (eg, if someone enters their DOB as September 10, 1986 instead of September 10, 1989, this may or may not be an indication of fraudulent reporting). Although personal information verification requires considerable staff time and effort, our results support its continued implementation.

When implementing the verbal identity confirmation check, our team did not expect many participants to fail it. However, our results suggest that this check was surprisingly effective at identifying fraudulent participation attempts. Anecdotally, most failures of the verbal identity confirmation occurred when participants refused to provide identifying information when prompted or simply hung up the phone without responding. One possible explanation for the unexpected utility of this active check is that it is the first time participants are prompted in real time to verify their identity. Because administering a verbal check can be completed remotely over the phone relatively quickly, we recommend the use of this check when possible.

In accordance with previous research, the consistent reporting review was also effective at identifying fraudulent or poor-quality submissions [34,35,44,45]. In particular, this active check acted as a catch-all as the final step of the authenticity protocol before the longitudinal portion of the study. Of note, reviewing data from multiple study time points is time consuming. Research teams may elect to adapt decision rules for when to conduct this check to increase feasibility.

Overall, the active checks appeared feasible to implement. Creating clear protocols for administering and interpreting each check reduced staff burden and increased standardization. Throughout the course of the study, we attempted to increase automation of these checks to further reduce staff burden, such as automatic IP address verification and automatic logging of attention check outcomes. Study teams conducting similar research may elect to implement some or all of these checks to tailor to their specific research goals, as well as further automate to reduce required staff effort.

### Data Integrity and Third-Party Recruitment Companies

Importantly, the results of this report suggest that previously established strategies for participant authentication can be successfully adapted and integrated for use in conjunction with web-based recruitment companies. However, as with all web-based or remote recruitment efforts, our results suggest that working with a third-party recruitment company to manage and tailor an advertising campaign is not enough to ensure data integrity. A sizable proportion of research participants identified by the company our team worked with did not pass authenticity checks and were withdrawn from the research study. However, this proportion of fraudulent submissions may still be lower than what is expected from other web-based recruitment sources [36].

### Authenticity Protocols and Barriers to Participation

Consistent with findings from other web-based studies, our results suggest that at least some fraudulent or inconsistent research submissions can be identified primarily using information provided as part of regular participation (eg, survey responses) [33,34,44,45]. We considered several additional authentication strategies for this study that were eventually deemed to be too high of a risk for introducing substantial bias into our sample, including requiring video sessions (ie, inaccessible for individuals without video-capable devices or reliable internet service), requiring photo ID (ie, inaccessible for individuals without this form of ID), and verifying address using physical mail (ie, time consuming and excludes individuals who are unstably housed). The ability to identify these invalid responses with information already typically required of participants is promising and may help to ensure that a broad spectrum of experiences is represented in the research. Research teams must weigh the added burden to participants and potential addition of barriers to participation when selecting authenticity strategies to limit sampling bias.

### Strengths and Limitations

The primary strength of this report is the formative approach to establishing an authenticity verification system for use in the context of remote recruitment, particularly openly recruited through online advertisements that are particularly vulnerable to fraudulent attempts to enroll in research studies. An additional strength is the effort to consider and balance the goals of maximizing data integrity and validity while minimizing barriers to participation (eg, requiring photo ID or an in-person study visit). Despite the introduction of this protocol, participants recruited remotely were only required to have a valid email address and text-capable phone, and the verification procedures did not unduly increase the time or effort of participation. The use of active authentication checks has also allowed our team to continue to improve our authentication system over time, as we developed many of our checks in response to ambiguous or nuanced situations we encountered.

However, as with any in-depth protocol, implementing an authentication system requires some staff effort. Authentication methods that are more likely to introduce barriers to participation may require less staff effort, and consequently, study teams will need to continue to weigh the costs and benefits of the processes they choose based on resources and potential for bias within the study population of interest. In addition, strategies for automating some of the more time-intensive elements (eg, using an automatic data-driven approach to flagging data inconsistencies for closer inspection) may enhance the efficiency of such approaches.

The authentication steps themselves also have several limitations. First, regarding the personal information verification, it is possible that these methods restrict study sign-ups among family members or individuals living in the same household. Some households may share phone numbers or emails, and many more likely share networks and devices that can access the internet. IP address verification may be most effective when examining attempts for repeat responses close in time. Second, address verification (part of personal information verification) is reliant on publicly available information. Newer construction or redistricting may not be accurately reflected in these datasets, causing the check to be inaccurate in rare cases. Third, our team encountered several instances wherein verbal identity confirmation was difficult to interpret. When prompted to provide their information, some individuals would hang up the phone, pause for a long time before responding, or call back later to provide the information. Many instances of failure for this check were more ambiguous than for other checks, requiring our team to establish the strict definition of failure reported when prompted. Fourth, we developed cutoffs for inconsistency reporting (part of consistent reporting review) based on preliminary data from approximately 30 individuals who had already passed all other checks. Because some room for error is to be expected in self-report data, particularly in TLFB interviews when participants are asked to recall events of up to the past 30 days, our selected cutoff of 4 inconsistencies should not be interpreted as a standard. Fifth, the introduction of automatic SMS verification for participants recruited remotely may prevent individuals without text-capable devices (eg, landlines) from joining the study. However, participants recruited through more traditional methods (eg, EHR review) are still able to participate without a text-capable phone.

### Conclusions

The authentication protocol presented, including both passive and active authentication strategies, provides an example of how research teams can increase confidence in the validity of data collected from remotely recruited research participants. Importantly, the system displays how authentication can occur while minimizing the introduction of barriers to participating in research, which may be of particular concern when conducting research with populations that have been historically marginalized or on highly stigmatized health behaviors (eg, opioid-involved PSU). Initial descriptive data indicate that web-based methods may be particularly effective for recruiting participants reporting PSU and other populations that have been highly stigmatized and that working with third-party web-based recruitment companies can be valuable for tailoring advertisement campaigns. Considering the utility of different authentication strategies, those that examine responses for inconsistencies across study activities may be particularly effective (ie, personal information verification, verbal identity confirmation, and consistent reporting review). Protocols to detect fraudulent or inconsistent reporting are important for assessing the validity of data collected remotely, and research teams will need to continue to adapt and tailor these strategies as remote recruitment methods evolve.

## Appendix

Multimedia Appendix 1 Remote recruitment identity authenticity protocol.

## Abbreviations

DOB: date of birth
EHR: electronic health record
MAR: missing at random
PI: principal investigator
PSU: polysubstance use
TLFB: timeline follow-back

## Data Availability

The datasets generated or analyzed during this study will be available in the Helping to End Addiction Long-term Data Platform after the conclusion of study activities [57].
