# Supplementary material for: Improving Data Integrity in Samples Obtained From Web-Based Recruitment: Protocol for the Development of a Novel System for Assessing Participant Authenticity in a Remote Longitudinal Cohort Study of Polysubstance Use
Source: JMIR Res Protoc. 2025 Aug 14;14:e69956. doi: 10.2196/69956 (PMC12395112; doi:10.2196/69956)
Supplement: Multimedia Appendix 1 [file resprot_v14i1e69956_app1.docx]

**Multimedia Appendix 1.** Remote recruitment identity authenticity protocol.^a^

| Study flow and check | | Active versus passive | Date added to study protocol | Purpose | Steps to implement | Interpreting the results of the check |
| --- | --- | --- | --- | --- | --- | --- |
| **During interest form** | | | | | | |
|  | Email or SMS verification | Passive | January 29, 2024 (email); May 8, 2024 (SMS) | Prevent bots from participating; certify that email and phone numbers are valid and accessible by the participant. | This strategy is implemented by a third-party partner. | Individuals who do not verify their email or phone number cannot complete the interest form. |
|  | Web page URL updates | Passive | February 28, 2024 | Reduce the likelihood of dissemination of the study landing page for organized fraud. | This strategy is implemented by a third-party partner. | Individuals using an outdated version of the web page cannot complete the interest form. |
|  | IP address restrictions | Passive | February 29, 2024 | Prevent submissions from outside of the intended study population and reduce the likelihood of completion of multiple submissions under different names. | This strategy is implemented by a third-party partner. | Individuals with IP addresses outside of the United States or who have already been used to sign up are blocked from viewing and completing the interest form. |
| **After interest form** | | | | | | |
|  | Interest form duplication review | Active | March 14, 2024 | Identify participants attempting to participate under different names and ensure a single participant is not identified by both remote and nonremote recruitment streams. | Study staff use search terms to examine participant records for duplicates. | Potential participants fail if the names, addresses, or contact information they provide on their interest form match the information provided on a different interest form. In such cases, neither individual is invited to continue participation. |
| **During screening survey** | | | | | | |
|  | Randomized survey passwords | Passive | March 6, 2024 | Reduce the likelihood of participants completing multiple screening surveys due to guessing another participant’s password or incorrectly inputting their own. | Participants are assigned a random 10-digit password that they must input to progress with study assessments; passwords are generated and stored in study logs. | Individuals who attempt to guess passwords to take assessments multiple times will have a very low likelihood of doing so. |
|  | Attention check | Active | May 16, 2024 | Identify inattentive participants; identify participants responding “yes” to all items they perceive to be inclusion criteria. | Attention check item is built into the screening survey such that study staff are alerted when a submission does not pass. | Participants fail if they respond incorrectly to the attention check item. |
| **After screening survey** | | | | | | |
|  | Personal information verification | Active | March 14, 2024 | Identify inconsistencies in name, contact information, address, and other information. | Study staff compare responses on identifying information across time points. | Participants fail if they provide differing names, addresses, or contact information at screening compared to the interest form. |
| **During baseline assessment** | | | | | | |
|  | Real-time survey or interview requirement | Passive | N/A^b^ | Provide the study team with the opportunity to verify a participant’s identity in real time, verify participant access to their reported phone number, and ensure participant willingness to complete real-time assessments. | Study was designed to require a real-time assessment before enrollment. | Participants who refuse to complete a real-time phone-based assessment cannot progress in the study. |
|  | Verbal identity confirmation | Active | April 16, 2024 | Require participants to produce identifying information in real time. | Before beginning their first phone-based assessment, study staff prompt participants to state their name, DOB^c^, and zip code. | Participants fail if they are not able to provide verbal confirmation of their identifying information in a timely manner. |
| **After baseline assessment** | | | | | | |
|  | Consistent reporting review | Active | April 23, 2024 | Identify inconsistent reporting of demographic information and study variables (eg, substance use patterns) between screening and baseline assessments. | Study staff review DOB consistency across assessments; if deemed necessary, study staff examine dates of completion of study assessments to determine which variables overlap and then examine for inconsistencies. | Participants fail if there are ≥4 inconsistencies identified by study staff. |

^a^Multimedia Appendix 1 lists each passive and active authenticity check described in the text, when in the study flow the check is administered (or automated), what the check is attempting to prevent, steps taken to implement for this specific study, and how our research team conceptualized passing or failing the check. It must be noted that the steps necessary to implement a given check for a different study may be different from those listed here.

^b^N/A: not applicable.

^c^DOB: date of birth.
